# ROS Pleiotropy in Melanoma and Local Therapy with Physical Modalities

**DOI:** 10.1155/2021/6816214

**Published:** 2021-11-03

**Authors:** Sanjeev Kumar Sagwal, Sander Bekeschus

**Affiliations:** ZIK Plasmatis, Leibniz Institute for Plasma Science and Technology (INP), Felix-Hausdorff-Str. 2, 17489 Greifswald, Germany

## Abstract

Metabolic energy production naturally generates unwanted products such as reactive oxygen species (ROS), causing oxidative damage. Oxidative damage has been linked to several pathologies, including diabetes, premature aging, neurodegenerative diseases, and cancer. ROS were therefore originally anticipated as an imperative evil, a product of an imperfect system. More recently, however, the role of ROS in signaling and tumor treatment is increasingly acknowledged. This review addresses the main types, sources, and pathways of ROS in melanoma by linking their pleiotropic roles in antioxidant and oxidant regulation, hypoxia, metabolism, and cell death. In addition, the implications of ROS in various physical therapy modalities targeting melanoma, such as radiotherapy, electrochemotherapy, hyperthermia, photodynamic therapy, and medical gas plasma, are also discussed. By including ROS in the main picture of melanoma skin cancer and as an integral part of cancer therapies, a greater understanding of melanoma cell biology is presented, which ultimately may elucidate additional clues on targeting therapy resistance of this most deadly form of skin cancer.

## 1. Introduction

Reactive oxygen species (ROS) are a consequence of imperfect aerobic metabolism. ROS are formed as a byproduct of electron transfer reactions from enzymatic and nonenzymatic sources [1]. This review also uses the term ROS to cover reactive nitrogen species (RNS) as many contain (reactive) oxygen. Oxidative stress is caused by the increased ROS or decrease in the activity of antioxidant systems in the cell. Slightly or detrimentally higher ROS levels have been coined oxidative eustress and oxidative distress, respectively [2]. The former relates to lower concentrations of ROS that amplify physiological processes such as proliferation and wound healing [3]. The latter covers exceedingly high levels of oxidative stress-provoking damage and cell death. Oxidative stress has been involved in several pathophysiological conditions, including cancer, by damaging lipids, proteins, and DNA [4–8]. ROS interaction with proteins impacts several signaling pathways by oxidizing redox-reactive cysteine and tyrosine residues within or nearby active sites [9, 10]. The effects of ROS vary from reversible to irreversible depending on the ROS levels and antioxidant machinery efficiency in the cells. Milder effects of protein modifications are reversible and promote cellular signaling through a change in protein activity [7]. For example, while irreversible modification of cysteine residues in proteins can lead to permanent loss of protein function, reversible modification can be protective from excessive ROS [5]. Adaption to ROS elevation through protein modifications plays a prominent role in ROS metabolism either by activating antioxidant pathways (e.g., Kelch-like ECH-associated protein 1 (KEAP1)) through cysteine residue modification or metabolic pathways (e.g., pyruvate kinase isoenzyme type M2 (PMK2)) [9]. Other reversible modifications, including glutathionylation, S-sulfonation, CoAlation, nitrosylation, and disulfides, can modify proteins by protecting them from terminal oxidation and alter their functions to adapt to oxidative stress [5]. Subsequently, ROS-dependent signaling modulates the activation of transcription factors such as NF-*κ*B (nuclear factor kappa-light-chain-enhancer of activated B-cells) and AP-1 (activator protein 1) [11–13]. Numerous mechanisms through which melanoma cells limit ROS exposure have been described. For instance, NRF2 (nuclear factor erythroid 2–related factor 2) is the most ubiquitous transcription factor that regulates genes involved in antioxidant defense [14]. Hypoxia and activation of oncogenes can induce NRF2, with evidence that this response is mandatory for melanoma development [15, 16]. Thus, the influence of ROS on cellular process is complex and they have dual role of pro- and antitumorigenic effects depending on their regulation.

Based on the work done by several groups, melanoma is a ROS-operated tumor [17]. The contribution of ROS to melanoma therapy is multifaceted. In melanoma prevention studies, antioxidants failed to show any beneficial effects. In some instances, an increase in cancer development was even observed under antioxidant supplementation [18, 19]. Many studies have shown that increased oxidative stress results in increased sensitivity of cells to therapy-induced cell death [20]. Survival of cells under the burden of oxidative stress depends on activating ROS scavenging pathways that are not needed in normal cells, deducing that interference with these antioxidant pathways or additional ROS burden may selectively kill melanoma cells [20, 21]. Impressively, several commonly used chemotherapeutic agents and physical modalities effectively induce ROS as part of their mechanism of action [21–23]. Conversely, ROS may also affect the outcome of immunotherapy [24]. For instance, chimeric antigen receptor (CAR) T cells are prone to hostile inflammatory conditions [25]. Hence, appropriate combinatorial approaches are essential to overcome therapy resistance and achieve better efficacy in melanoma therapy. This review discusses the origin and types of ROS. Further, their signaling and damaging effects in melanoma initiation and progression are described. Moreover, several physical treatment modalities are summarized that contribute to local ROS production and subsequent antimelanoma efficacy

## 2. ROS: Types, Sources, and Regulation

ROS are molecules and free radicals involved in the transfer of electrons from reactive oxygen. There are various tools for indirectly measuring ROS in cells and tissues while measuring ROS directly is still deficient. A selection of ROS is described hereafter, which are relevant in both physiology and pathology, including cancer.

### 2.1. ROS Types

**Figure 1:**
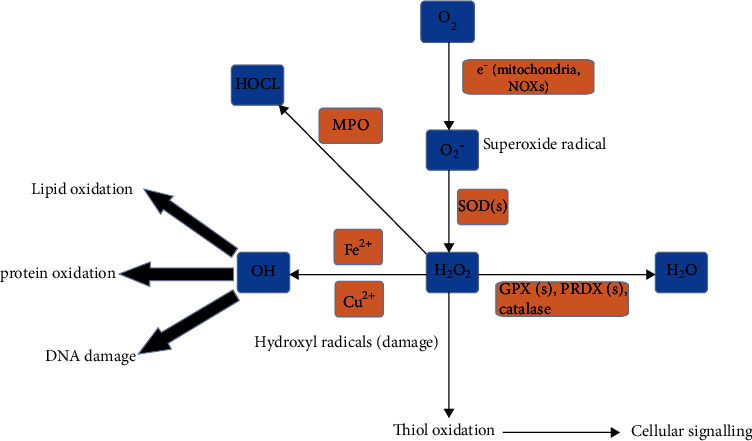
ROS types and generation. H_2_O_2_ and O_2_^−^ are released by mitochondria into the cytosol. SODs convert O_2_^−^ in the cytosol to H_2_O_2_. NADPH oxidases (NOXs) also generate O_2_^−^ in the cytosol. H_2_O_2_ is converted to H_2_O by GPXs and PRDXs. The reaction of ferrous or cuprous ions with H_2_O_2_ forms OH· radicals, subsequently damaging lipids, proteins, and DNA. H_2_O_2_ affects signaling through oxidations of protein thiols. Abbreviations: GPXs: glutathione peroxidases; PRDXs: peroxiredoxins; SODs: superoxide dismutases. Created with http://biorender.com.

**Figure 2:**
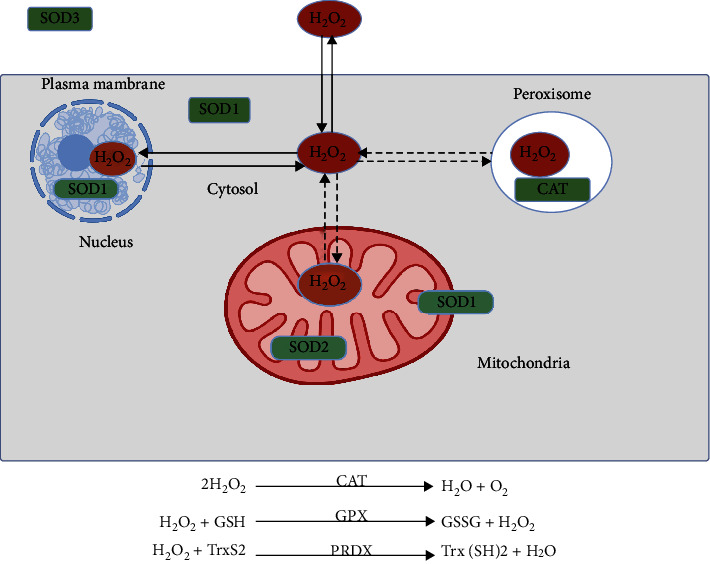
ROS detoxification. The cell is equipped with defense mechanisms to scavenge ROS. Detoxification enzymes like CAT react with H_2_O_2_ to catalyze the formation of H_2_O and O_2_. GPX and PRDX reduce H_2_O_2_. Abbreviations: CAT: catalase; GPX: glutathione peroxide; PRDX: peroxiredoxins; GSSG: glutathione disulfide, oxidized; GSH: glutathione, reduced. Created with http://biorender.com.

**Figure 3:**
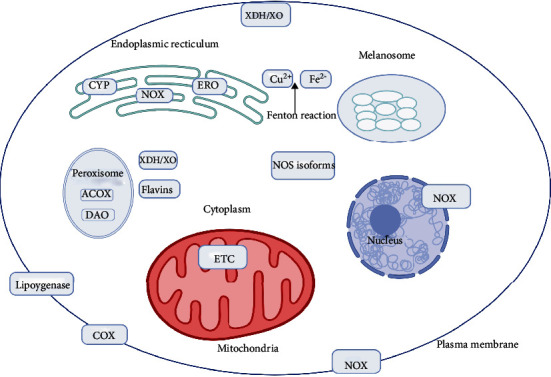
Sources of cellular ROS production. Prominently, melanosomes, mitochondria, and NOXs and NOS uncoupling generate ROS. Primarily, NOXs are localized in the plasma membrane, although they can be found on other membranes as well, including the endoplasmic reticulum and mitochondria. Cytosolic enzymes such as XO, XDH, and soluble components like flavin contribute to intracellular ROS production. Oxidative protein folding by ERO1 and enzymes like CYP in the endoplasmic reticulum and lipoxygenase and cyclooxygenase in the plasma membrane also generate intracellular ROS. Peroxisomes containing DAO and ACOX generate ROS, too. Abbreviations: XO: xanthine oxidase; XDH: xanthine dehydrogenase; ERO1: endoplasmic reticulum oxidoreductin; CYP: cytochrome P450-dependent monooxygenases; DAO: D-amino oxidase; ACOX: acyl-CoA oxidase. Created with http://biorender.com.

The primary production site of superoxide is mitochondria (Figure 1). During the leakage of electrons at several respiratory chain respiratory complexes, especially complex I and III, molecular oxygen is reduced by one electron to produce superoxide anion (O_2_^−^). O_2_^−^ is a moderately reactive short-lived species that dismutates spontaneously or by superoxide dismutases (SOD) (Figure 2) to H_2_O_2_ [26]. This type of ROS is generated by the autoxidation of various small molecules such as dopamine, flavins, and hydroquinones (Figure 3). It is produced nonenzymatically when prosthetic groups or reduced coenzymes directly transfer a single electron to oxygen. Enzymatically, NADPH oxidases (NOXs) reside on the cell membrane of many cell types to produce extracellular superoxide [27]. Superoxide releases iron by targeting iron-sulfur (Fe-S) clusters or react with nitric oxide (NO·) to form peroxynitrite (ONOO^−^) [26]. ONOO^−^ is a strong oxidant that indiscriminately reacts with DNA to generate double-stand breaks, oxidation of amino acids in proteins and induces lipid peroxidation by reacting with lipids.

Hydrogen peroxide (H_2_O_2_) plays a role as a second messenger in several pathways [28] by oxidizing the thiol group (-SH) on cysteine residues, resulting in the transduction of extracellular and intracellular signals and control of the gene expression [29]. Cysteine residues exist as thiolate anion (Cys-S-) at physiological pH and are more susceptible to oxidation compared with the protonated cysteine thiol (Cys-SH) [9]. Reversible modifications like sulfenic species are generated after enzyme-mediated oxidation of cysteine residues by H_2_O_2_ and can be returned to reduced states by the action of thioredoxin (TRX) and glutaredoxin reductases (GRX) [30]. However, advanced oxidation to sulfinic acid and irreversible oxidation to sulfonic acid results in permanent damage of protein function [30]. Cells have professional enzymes directed to prevent the buildup of intracellular H_2_O_2_, primarily peroxiredoxins (PRDXs) and glutathione peroxidases (GPXs) [30]. To decrease O_2_^−^-mediated ONOO^−^ formation (Figure 2), SOD1 (located in the cytoplasm and mitochondria) and SOD2 (located in the mitochondrial matrix) convert O_2_^−^ generated by mitochondria and NOXs into H_2_O_2_ [30]. H_2_O_2_ diffuses freely to other sites in or outside the cell. When present in peroxisomes, catalase (CAT) can react with H_2_O_2_ to form water and oxygen. The endoplasmic reticulum is another primary source of H_2_O_2_ that is generated by the combined action of protein disulfide isomerase (PDI) and ER oxidoreductin 1 (Ero 1) during the formation of disulfide bonds [31]. It is noticeable that the concentration of H_2_O_2_ defines its role as a signaling molecule (1-10 nM) regulating kinases and phosphatase-driven pathways or causes oxidative stress (>100 nM) [28].

Hydroxyl radicals (OH·) are the most reactive type of ROS. It is generated in the Fenton reaction with H_2_O_2_ [32], instigating lipid peroxidation and commencing lipid radicals and lipid peroxyl radicals [33]. These short-lived radicals initiate lipid peroxidation by reacting with hydrogen atoms of polyunsaturated fatty acids, which are highly reactive because of the double bonds between carbon atoms in those fatty acids. Lipid peroxidation and GPX4 regulate an iron-dependent cell death known as ferroptosis, relevant to normal and pathological processes [34]. Several other transition metal ions can also react with H_2_O_2_ to produce peroxyl and alkoxyl radicals [32]. The reduction of Fe^3+^ to Fe^2+^ by superoxide ion also leads to OH^·^ under specific conditions [35].

### 2.2. ROS Sources

There exist several sites inside a cell that generates ROS. A large share of intracellular ROS is produced in the electron transport chain (ETC) [9]. ROS generated by the ETC into the mitochondria can be delivered into the cytoplasm through the permeability transition pore (PTP) [29]. The opening of PTP leads to a decrease in the concentrations of ATP and Ca^2+^ to release cytochrome c [36–38]. This fuels the collapse of the membrane potential of mitochondria and a sudden increase in ROS generation by ETC [39]. It has been affirmed that this results in autophagy and apoptosis or necrosis, depending upon the extent of organelle damage. Hence, mitochondria are a major site for ROS production, especially complexes I and III [40, 41]. In addition to mitochondria, peroxisomes and the endoplasmic reticulum produce ROS. Peroxisomes contain several H_2_O_2_ generating enzymes, and catalase (CAT) in this organelle detoxifies several substrates and toxic molecules (Figure 2). Other ROS-generating enzymes include xanthine oxidase, *α*-ketoglutarate dehydrogenase complex, and NOXs [42]. Apart from phagocytes, NOXs are also found in nonphagocytic cells that regulate cellular growth responses [42]. It has been noticed that ROS produced by NOXs enhances melanoma cell proliferation through activation of NF-*κ*B [43]. The oxidase components are expressed by both melanoma cells and melanocytes [43]. In primary and metastatic melanoma cells, higher levels of NOX and oxidative have been found compared to normal human melanocytes [44]. ONOO^−^, a species generated by the interaction of NO· with NOX-generated O_2_^−^, is highly reactive toward redox-sensitive amino acid residues, including cysteine and tyrosine [42]. Enzymes like NO· synthases (NOS), xanthine oxidoreductase, and cytochrome c oxidase can be involved in NO· production [45].

### 2.3. ROS Regulation

**Figure 4:**
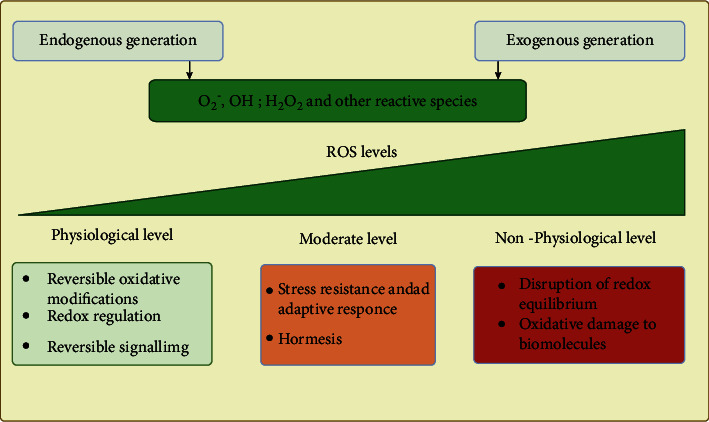
Cellular responses to endogenous and exogenous ROS. The response gradient will vary with the cell type, the location of ROS source inside the cells, and the activity of detoxifying enzymes. Blue and red colors represent predominantly beneficial or deleterious responses to ROS levels. Created with http://biorender.com.

**Figure 5:**
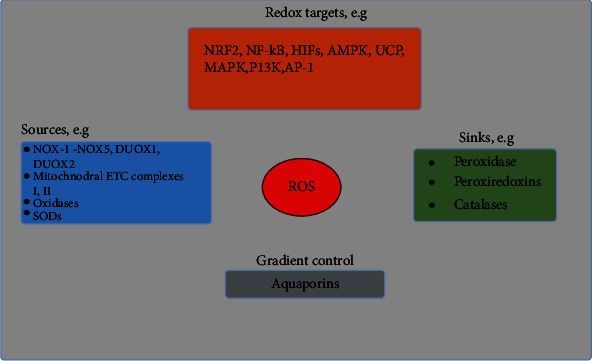
Targets and modulators of ROS. Sources of ROS (blue), its targets (orange), aquaporins (grey), and sinks (purple) are shown. Redox targets serve as the center point to support biological functions. SODs serve as a source for ROS and as a sink for the O_2_^−^. Under homeostatic conditions, moderate or low levels of ROS induce the activation and inactivation of transcriptions factors. These transcription factors regulate stress adaptation (including antioxidant response), inflammatory response, hypoxic response, metabolic adaptation, and cell death. Abbreviations: SODs: superoxide dismutases.

To prevent ROS overload in the cytosol and ER, cells have antioxidant defense mechanisms in place tightly regulating ROS levels and maintaining the reduced state of critical biomolecules (Figure 4). Reduced glutathione (GSH) is the prolific reducing agent in the cytosol and ER, though the ratio of reduced to oxidized (GSSG) glutathione (GSH: GSSG) varies in these two compartments [46]. GSH diffuses from the production site to distant sites by passing through the membranes and plays a prominent role in the ROS detoxification in cancer cells [47]. GSH is a glutamate, glycine, and cysteine tripeptide, synthesized by two enzymatic steps catalyzed by glutamate-cysteine ligase (GCLC) and GSH synthetase (GSS) to from the tripeptide. GSH is used by GSH peroxidases (GPXs) and GSH S-transferases (GSTs) for the elimination of ROS [48]. In addition to GSH-dependent antioxidant systems, there is another less abundant small protein antioxidant system consisting of PRDXs, having a high catalytic activity toward H_2_O_2_ and being rejuvenated by thioredoxin (TXN) and sulfaredoxin (SRX) networks [49]. The oxidized forms of TXN and GSH are rejuvenated by TNX reductases [50] and GSH reductases (GR), respectively, using NADPH as an electron donor [51]. Oxidative stress develops when the production of ROS outpaces the scavenging ability of the cellular defense system made up of redox enzymes and several other antioxidant molecules [52]. Oxidative stress promotes the expression of enzymes involved in TXN and GSH systems, implying that they work in accord to buffer the stress induced by ROS molecules [53]. TXN is less significant as an antioxidant due to its lower concentration in cells (*μ*M compared to mM range of GSH), controlling the redox state of specific factors by performing rapid oxidation-reduction reactions kinases and transcription factors [53]. In addition, GSH can reverse modifications such as sulfenylation mediated by ROS. In summary, several ROS sources affect redox targets and are additionally controlled by ROS sinks, while melanoma cells showed altered expression among all those steps (Figure 5).

## 3. Pleiotropic Roles of ROS in Melanoma Biology

Melanocytes are sensitive to oxidative stress induced by an imbalance of ROS. In normal melanocytes, melanin acts as an antioxidant and suppresses H_2_O_2_, O_2_^−^, and singlet oxygen [54]. However, melanogenesis in melanoma cells itself is a source of ROS and oxidative stress [55]. O_2_^−^ and diffusible H_2_O_2_ produced by the mitochondria and NOXs play an important role in melanocyte malignant transformation [27]. Such Oxidative stress can cause an imbalance of homeostasis in melanocytes, jeopardizing their survival or leading to malignant transformation. In melanoma, NOX4 is highly expressed as compared to the low levels in melanocytes [56]. Overexpression of NOX1, uncoupled eNOS, and NOX4 produced ROS in melanoma, linked to the epithelial-mesenchymal transition [56, 57]. Uncoupled eNOS contributes to superoxide production during malignant transformation [57]. NOS-dependent superoxide formation has a prominent role in melanoma genesis [57]. Furthermore, these data show that superoxide production by eNOS plays a prominent role in melanoma cells' survival and melanocyte malignant transformation. In addition to that, melanoma cells require NADPH generating enzymes to promote distant metastasis. H_2_O_2_, O_2_^−^, and singlet oxygen are involved in various stages of melanomagenesis and tumor microenvironment (TME) quality, including hypoxia, metabolic profiles, immune responses, biosynthesis of melanin, metastasis, and oxidative profiles. ROS increases the toxicity of RNS dramatically begins melanomagenesis that attributes to the leakage of melanosome contents. Several findings suggested that worsen oxidative stress leads to the mutation in several melanoma-associated genes. For example, the somatic BRAF V600E mutation in melanoma can be induced by oxidative stress [58] and loss of p16 results in elevated ROS and mitochondrial biogenesis of human melanocytes [59]. In addition, melanoma progression is associated with depletion of PTEN and the resulting increase in O_2_^−^ [60]. Hence, oxidative stress is a driver of melanomagenesis.

### 3.1. Antioxidant Network

The role of antioxidant systems is dual in melanoma initiation, progression, and metastasis. The misbalanced activation of the antioxidant transcription factor NRF2 leads to the promotion of melanoma [61]. This transcription factor is entangled in transcribing several GSH and TXN antioxidant pathways genes under various physiological and pathophysiological conditions [62]. In basal conditions, the level of NRF2 is under control by its association with KEAP1, which promotes its degradation via the ubiquitin-proteasome pathway. Increased ROS levels lead to oxidative stress modifications of cysteine residues of KEAP1, leading to flawed NRF2 ubiquitination and NRF2 accumulation [61]. Several mechanisms exist for NRF2 accumulation, such as mutations in the KEAP1 and NRF2 genes, carcinogen-induced DNA damage, and inactivation of KEAP1 due to methylation of its promoter [63]. The role of NRF2 is convoluted and tissue dependent. Loss of NRF2 bolsters epithelial-mesenchymal transition through ROS to promote migration and invasion to support invasion and diapedesis of cancer cells [64, 65]. In contrast, NRF2 can uphold migration and invasion through the BACH1 (BTB domain and CNC homolog 1) transcription factor [66]. Therefore, the role of ROS and NRF2 is complex at different tumor stages. Other agents involved in antioxidant defense and H_2_O_2_ removal include GPXs, PRDXs, and CAT [12]. The antioxidant capacity of melanoma differs from normal melanocytes and that of other skin cancers. The expression and activity of the antioxidant enzymes catalases, Mn-SOD2, and Cu/Zn-SOD1 are higher in melanoma than in basal cell carcinoma and squamous cell carcinoma [67]. This explains that increased oxidative stress is an important marker in melanoma development. Furthermore, the GSH: GSSG ratio is also higher in melanoma compared with the other skin tumors. This suggests that in melanoma, the increased levels of GSH can readily scavenge ROS and that the subsequently formed GSSH is efficiently reduced to GSH. Collectively, these data imply that melanoma has a better antioxidant status than other skin tumors. The increased resistance of melanoma cells to oxidative stress is not observed in melanocytes [68], suggesting that acquiring an elevated antioxidant network is critical for melanoma development. In primary melanoma, Mn-SOD2, Cu/Zn-SOD1, and CAT expression are elevated compared with normal skin and melanocytic nevi [67]. In addition, melanoma metastases show improved resistance to oxidative stress and display high levels of ferritin expression compared with their corresponding primary melanomas [69]. Ferritin binds to and prevents iron from being reduced in the Fenton reaction, thereby averting OH·-induced lipid peroxidation and apoptosis [69]. Summative, these findings indicate that primary and metastatic melanomas are highly resistant to oxidative stress through the increased activity of several antioxidative mechanisms. In other words, inhibition of ROS by antioxidants does not have a predictable outcome on cell function since the role of ROS changes under differing environmental conditions. For future aspects, it will be essential to identify specific molecular targets of ROS under different conditions to modulate pathways downstream of ROS that increase adaptation to stress to increase therapeutic efficacy.

### 3.2. Apoptosis

In response to ROS, melanoma cells can, in principal, succumb to regulated cell death. However, in approximately half of the sporadic melanomas, Protein kinase B, also known as AKT, is hyperactivated because of gene amplification and decreased PTEN (phosphatase and tensin homolog) activity [70]. Activated AKT can subsequently phosphorylate and thereby inhibit the activity of the proapoptotic factors BAD (Bcl-xL/Bcl-2-associated death promoter), caspase (cysteinyl-aspartate specific protease) 9, forkhead transcription factor, GSK3 (glycogen synthase kinase-3), and IKK (inhibitor of NF-*κ*B). AKT stabilizes cells with extensive mitochondrial damage, which can generate surplus ROS [60]. Furthermore, AKT induces the expression of the ROS-generating enzyme NOX4 in melanoma cells and growth melanoma cells in mice [60]. In addition, the RAS/BRAF/MEK/ERK mitogen-activated protein kinase pathway is constitutively activated in melanoma via an activating mutation in BRAF or autocrine growth factor stimulation [71] and is a crucial modulator of melanoma initiation and progression [72]. Mitogen-activated protein kinases regulate ROS production by melanoma cells and cooperate with antiapoptotic proteins to maintain melanoma cell viability [73]. ROS constitutively activate NF-*κ*B [74], a transcription factor critically involved in cell survival [75]. The activation of NF-*κ*B has been proposed as an event that promotes melanoma tumor progression [76]. Transcription activation of NF-*κ*B-regulated chemokines enhances melanoma progression through autocrine and paracrine loops, resulting in autonomous growth and invasion of melanoma cells [77]. Furthermore, ROS can activate AP-1 [78], a transcription factor critically involved in RAS-induced oncogenic transformation [79]. Furthermore, ROS regulate the expression of matrix metalloproteinase (MMP)1, MMP2, and urokinase plasminogen activator (uPA) [80, 81]. These proteinases are highly expressed in melanoma [82] and contribute to their migratory capacity. Recently, ROS-induced apoptosis of melanoma cells was shown to contribute to vasculogenic mimicry [83]. This process mimics the activity of endothelial cells and results in the formation of a fluid-conducting, matrix-rich meshwork [84] that contributes to melanoma progression [85, 86]. As such, the proapoptotic activity of ROS contributes to melanoma progression. ROS' proapoptotic and antiapoptotic effects in melanoma cells appear to be a driving force of melanoma development.

### 3.3. Hypoxia

The role of mitochondrial ROS in apoptosis and hypoxia-induced gene transcription has been elucidated recently [15]. The well-established role of mitochondrial ROS for the stabilization of hypoxia-inducible transcription factors (HIFs) under hypoxia leads to angiogenesis through the upregulation of vascular endothelial growth factor (VEGF) expression [87]. The epidermal component of normal skin in which melanocytes reside is a mildly hypoxic environment, predicted due to the distance between the skin and superficial blood vessels [88]. To counteract the adverse effects of low oxygen levels, HIFs activate gene expression regulating multiple biological processes, including metabolism, proliferation, apoptosis, and migration [89]. HIF-1 regulates most of the hypoxia-responsive genes [90]. The transcription factor consists of a constitutively expressed *β*-subunit and an oxygen-monitored *α*-subunit. Hence, HIF-1 is the master activator for dozens of target genes transcribed by cells in response to low oxygen concentrations [91]. HIF-1 activation is also required for the AKT-mediated transformation of melanocytes, hence regulating apoptosis [88].

Studies have identified increased HIF-1 expression and activity in melanoma under normoxia mediated by ROS and NF-*κ*B [92, 93]. Under normoxic conditions in nonmalignant cells, HIF-1 is rapidly degraded by the ubiquitin-proteasome system, and it is upregulated in a hypoxic microenvironment [94]. However, it has been recently reported that HIF-1 can be upregulated under normoxia in response to growth factors, hormones, cytokines, UV irradiation, and metal ions [95–97]. In addition, several HIF target genes are strongly expressed in melanoma already under normoxic conditions, and elevated HIF-1 activity was found in melanoma cell lines under normoxic conditions in contrast to other types of tumors. Immunohistochemistry of malignant melanoma showed focal expression of HIF-1 in cancer tissue independent of regional hypoxia [15, 89]. Furthermore, several studies have demonstrated that part of the normoxic expression of VEGF and AngPTL4 depended on HIF-1 [98]. Interestingly, incubation of melanoma cells under reduced oxygen tension did not lead to a more substantial upregulation otherwise found in nonmelanoma cells, supporting the high basal expression of HIF-1 under normoxia [98, 99].

Melanocytes are more prone to oncogenic transformation when grown in a hypoxic environment. The cells' primary function is delivering melanin in melanosomes to keratinocytes resulting in protection against the harmful effects of UV radiation [88]. Within the melanocytes, the synthesis of melanin results in the generation of H_2_O_2_ and, if inappropriately processed, OH· and other ROS [73]. In particular, melanosomes within melanoma cells are characteristically abnormal, with fragmented melanin and disrupted membranes. The disruption of melanosomal melanin is an early event in the etiology and progression of melanoma, leading to increased oxidative stress, ROS production, and DNA mutation [4, 6, 83]. Several studies revealed that such ROS are responsible for the increased HIF activity under normoxia in melanoma. The activity and protein level of HIF are strictly controlled by the quenching of ROS or inducing reagents. The crucial redox-sensitive transcription factors in mammalian cells are NF-*κ*B, NRF2, and AP-1 [74, 76]. ROS can activate the transcription factor NF-*κ*B that is constitutively activated in melanoma cells [74, 76]. NF-*κ*B, in turn, can induce HIF-1 expression and NF-*κ*B-HIF-1 interaction contributes to breast cancer metastatic capacity [88, 100]. Studies confirmed the regulation of NF-*κ*B through ROS in malignant melanoma and showed that the inhibition of NF-*κ*B by the adenoviral overexpression of the IKK led to the attenuation of the HIF activity [101]. These data support the concept of transcriptional regulation of HIF-1 by NF-*κ*B under normoxic conditions.

Besides the described ROS-dependent regulation, HIF-1 is translationally regulated by the mammalian target of rapamycin (mTOR). The mechanism of regulation of HIF by mTOR is poorly understood. It appears that under hypoxia, mTOR is inactivated, which led to the conclusion that mTOR signaling to HIF is oxygen independently regulated [102]. Under severe hypoxia, no influence of mTOR inhibitors was observed; thus, the stimulation of HIF-1 by mTOR is relevant under mild hypoxia or even normoxia only [102]. Several studies confirmed this hypothesis, as rapamycin reduced the HIF activity and protein expression under normoxia. One study showed that rapamycin, in contrast to ROS and NF-*κ*B, does not influence HIF-1 mRNA expression, suggesting posttranscriptional regulation. Recently, Aprelikova and colleagues described a novel role for the cancer-testis antigen melanoma antigen-11 (MAGE11) as an inhibitor of prolyl hydroxylase (PHD2) in hypoxic responses [103]. Strong expression of MAGE-11 has been seen in different melanoma cell lines, which led to HIF stabilization under normoxia [104]. The finding that the regulation of protein abundance and the transcriptional regulatory network is crucial in controlling HIF-1 levels in melanoma and other tumor types opens new therapeutic options in modulating HIF-1 activity.

### 3.4. Melanin Biosynthesis

Exposure to UV is suggested to be a significant risk factor for developing melanoma, especially during childhood [105, 106]. However, this only refers to the sun-induced type of melanoma and not other types of melanoma that were never exposed to the sun [107]. About 90%-95% of the solar UV radiation that reaches the earth is UVA. Due to its high penetration capacity, UVA can irradiate melanocytes even through clothes and windows [106]. In response to the direct mutagenic effect of UV radiation, melanin synthesis by melanocytes is induced. Although melanin is initially necessary for protection from UV, it can turn into a prooxidant under oxidative stress because of inflammation, UV exposure, or higher metabolic processes, thus regulating epidermal homeostasis and affecting melanoma behavior [108]. UV-induced melanin biosynthesis results in an increased cellular concentration of its reactive precursors. The initial reaction in melanin formation is the enzymatic oxidation of L-tyrosine to dopaquinone [109]. This reactive precursor is either converted into monomers that polymerize into black/brown eumelanin or reacts with a -SH group of cysteine to form 5-S-cysteinyldopa, ultimately forming the basic monomers for red/light brown phaeomelanin. Cysteine is a necessary amino acid for phaeomelanin production, even though it is also a part of the GSH molecule, which acts as a part of the defense system against intracellular ROS [110]. When cysteine is used for increased UV-induced production of phaeomelanin, less GSH is produced, and oxidative stress may be more likely. Oxidative stress, in turn, releases iron from its intracellular storages into the cytosol of mammalian cells [111]. In the presence of large amounts of iron, which is frequently observed in melanoma and its precursor stages but not in normal melanocytes [110], phaeomelanin and 5-S-cysteinyldopa become prooxidants [110]. Oxidized melanin reacts with O_2_ to form H_2_O_2_, O_2_^−^, and other radicals [104] and adversely affects ROS homeostasis. During the biosynthesis of phaeomelanin, 5-S-cysteinyldopa can disturb redox homeostasis directly through its ROS production in the presence of iron and indirectly through depletion of the GSH antioxidant buffer system. Their independent actions or dependent interaction play a role in UV-dependent or -independent melanomagenesis and progression and in drug resistance, as melanocytes and melanoma have higher ROS levels that seem to coevolve with enhanced antioxidant defense systems [112]. Thus, melanin and melanogenesis play a dual role in melanoma. They protect the melanocytes against insults, such as oxidative stress and UV radiation, but accelerate melanoma progression and weaken the effects of chemotherapy and radiation therapy [109, 113].

### 3.5. Metabolic Profile

Tumor cells are metabolically hyperactive, so it requires high ATP levels to enable cell proliferation. In melanoma, ATP is predominantly generated through aerobic glycolytic metabolic pathways and lactic acid production, which leads to several advantages for melanoma cells [114]. This includes, for instance, higher proliferation of tumor stem cell populations, increased hypoxia, elevated M2 macrophage polarization, lower intratumoral T cell activation, additional NADPH for ROS detoxification, and metastasis via MMP production. An in-depth analysis of adaptive redox homeostasis in melanoma and energy metabolism has been provided recently [115, 116], and the reader is referred to these and complementing views on oxidative phosphorylation [117].

### 3.6. Metastasis

Over the recent years, it has become evident that early inflammatory and angiogenic response and remodeling of the extracellular proteins are key factors (i.e., type I collagen) in creating a microenvironment that sustains tumor growth and metastasis [118]. Metastasis is a hallmark of most malignant tumors and the primary cause of mortality and morbidity in patients with melanoma [119]. The entry of tumor cells into the circulation is the critical rate-limiting step in metastasis that requires MMP expression [119], uroplasminogen activation, epidermal growth factor receptor-driven polarity changes and migration, interaction with integrins [120], and other mechanisms all tightly linked to ROS. The cytoplasmic TRX is a ubiquitous thiol-reducing system implicated in cancer progression of melanoma [121]. TRX can be bound by the endogenous inhibitor thioredoxin-interacting protein (TXNIP), which negatively regulates TRX [121]. Importantly, inhibition of TRX activity promotes the transendothelial migration (TEM) of melanoma cells in vitro through endothelial injury and the loss of VE–cadherin-mediated endothelial cell-cell adhesion [122]. Overexpression of TRX inhibits both the baseline and ROS-induced TEM. Therefore, ROS enhance the TEM of melanoma cells during intravasation, and XNIP and inhibition of TRX activity could trigger this mechanism. It has also been observed that hypoxia in melanoma xenografts induces a higher metastatic frequency by increasing the expression of hypoxia-inducible genes promoting metastasis in a radiated transplant animal melanoma model [122]. However, the regulation of intravasation in vivo is not simply a matter of high or low intracellular concentrations of ROS. It requires the coordinate expression and activity of, for example, IL-8-mediated chemotaxis and CD9 and the integrin-mediated adhesion of melanoma cells to vascular endothelial cells [122]. The expression of the genes that promote melanoma metastasis is upregulated after subcurative melanoma irradiation [122]. Intriguingly, antioxidant supplementation in vivo was observed to spur rather than inhibit melanoma metastasis in mice inoculated with melanoma cells individually isolated from patients [123]. The authors found that blood and viscera are especially impinging strong oxidative stress in melanoma cells, forming a natural barrier against cancer metastasis. Antioxidants hampered this barrier and thus allow melanoma cells that had migrated to the circulation to survive better, subsequently forming more metastasis. These results were re-iterated in a parallel in vivo study using the antioxidant N-acetyl-cysteine (NAC) [124].

### 3.7. ROS and Different Cell Types in the Tumor Microenvironment

The production of ROS by tumor cells plays a prominent role in driving tumorigenesis by shaping the tumor microenvironment (TME) [125, 126]. In addition, ROS generated by nontumor cells infiltrating the TME collectively decide the overall oxidative state of local TME. The TME consists of cancer cells, stromal cells, and immune cells. Immune cells infiltrate the environment of cutaneous melanoma during its early onset and throughout tumor development [127]. During inflammation, the migration of myeloid cells such as neutrophils, monocytes, eosinophils, and tissue-resident macrophages, dendritic cells, and mast cells play a role in cancer development [128].

Macrophages are of central importance in melanoma initiation and progression, especially tumor-associated macrophages (TAM) [129]. These cells fuel tumor growth by creating an immunosuppressive micro milieu via the production of chemokines, cytokines, and other mediators. Intriguingly, TAM polarization in the TME is associated with ROS and oxidative stress [130]. TAM, in turn, autoamplify ROS production via aberrant activation of NOX and NOS. The released species react to form ONOO^−^, a mutagenic agent that inhibits T cell activity [131]. This will affect cells nearby, their integrity, and the composition of the TME, such as matrix remodeling and angiogenesis [132]. In addition, this exerts selective pressure on the development of genetically adapted tumor cells with high resistance to oxidative stress pressure, affecting melanoma therapy [128]. It is also well established that ROS leads to metabolic reprogramming of different cell types in the TME [133, 134].

ROS-mediated metabolic reprogramming also changes the energy requirements of T cells in the TME [135, 136]. Effector T cells (T_eff_) are less oxidative and have more metabolic activity than naive T cells (T_n_). Naive T cells keep in check the ROS levels by persistently synthesizing antioxidant molecules to avoid excessive ROS, which otherwise would initiate cell death and introduce a constant prooxidative state in cancer cells [20, 137]. Activation of T cells is escorted by increases in glucose uptake and mitochondrial activity fueled by glutaminolysis [138, 139]. Studies have shown that low ROS levels generated by mitochondria are pivotal for NFAT (nuclear factor of activated T cells) activation and IL-2 production by T cells [140, 141]. By contradiction, ROS can selectively suppress the DNA-binding capacities of NF-*κ*B and NFAT, resulting in the downregulation of IL-2 transcription [142]. ROS being generated upon TCR engagement regulate ERK proliferative pathways and CD95/CD95L proapoptotic pathways, critical for normal T cell responses [143]. Hence, uncontrolled surplus ROS generation in the melanoma TME leads to nonfunctional T cells and failure to develop T_eff_ or T_m_ responses. Henceforth, ROS levels must be buffered in a safe range for clonal expansion and differentiation of an activated T cell through metabolic reprogramming. This is a daunting task, as antioxidants would improve T cell activity while at the same time also fueling melanoma growth.

In contrast to normal fibroblasts, which are responsible for the turnover of extracellular matrix (ECM), ROS-activated cancer-associated fibroblasts (CAFs) can be found at the edge of tumors or infiltrating the tumor [144–146]. These cells are a potent source of ROS, adding to the already hostile micro milieu [147]. Another considerable role of CAFs is to enhance tumorigenesis by activating specific signaling pathways crucial for promoting tumor growth. For instance, this is done through AKT in epithelial cells and the secretion of soluble factors like CXCL12 [148]. The domination of CAFs within cancer tissues is correlated with poor prognosis, elevated infiltration of tumor-associated macrophages, epithelial to mesenchymal transition, and ROS-driven hypoxia [149]. Hypoxia created by desmoplasia, in turn, stimulates the production of mitochondrial ROS, which can influence CAF function [150]. CAFs expressing smooth-muscle *α*-actin (*α*-SMA) are called myofibroblasts. The role of ROS in transition from fibroblasts to myofibroblasts is well reported, and this transition is driven by factors such as transforming growth factor beta1 (TGF-*β*1) and stromal cell-derived factor 1 (SDF-1) in a ROS-dependent manner [151]. Moreover, chronic oxidative stress also leads to the differentiation of fibroblasts to myofibroblasts. These ROS effects can be reversed with prolonged exogenous antioxidants in fibroblasts isolated from mouse models of oxidative stress that lack prominent antioxidant transcription factors [152]. In addition, antioxidant enzymes such as GPX3 and thioredoxin reductase I upregulation within fibroblasts inhibit differentiation into myofibroblasts. The conclusion of these observations establishes that ROS can enhance specific fibroblast subtypes, including the predominant myofibroblast differentiation in human tumors [153]. ROS produced by fibroblast can also augment tumorigenesis [154]. Numerous studies focused on the role of H_2_O_2_ in TME and stroma [155]. The H_2_O_2_ is produced by tumor epithelial cells and can diffuse to adjacent cells, inducing a more protumorigenic environment. This effect can be abrogated with the addition of CAT [155].

ROS levels are the prominent factor in deciding its role as a signaling molecule or oxidative stress-causing agent, leading to activation of various defense mechanisms or cell death [156]. ROS can regulate autophagy through LC3-associated autophagosomes or AMP-activated protein kinase (AMPK) and the regulation of gene transcription factor activity like NF-*κ*B inducing autophagy gene expression (BECLIN1/ATG6 or SQSTM1/p62) and unfolded protein response (UPR) during hypoxia [157]. Autophagy plays a complex role in the initiation of cancer [158]. Fibroblasts have p21^Ras^-independent ROS generating enzymatic systems which set up extracellular H_2_O_2_ in response to TGF-*β*1 [159]. In addition, an enzyme similar to 15-LOX in fibroblasts has been shown to procreate substantial amounts of O_2_^−^ that developed without flavoenzyme activity [160]. These modifications have a crucial impact on the proteins' signaling and functional role, augment genome instability, prevent inflammation, and make cancer cells survive under hypoxia and starvation [15]. In addition, elevation in autophagy is associated with metastasis and poor prognosis in melanoma patients [161].

## 4. ROS in Melanoma Therapy with Physical Modalities

**Figure 6:**
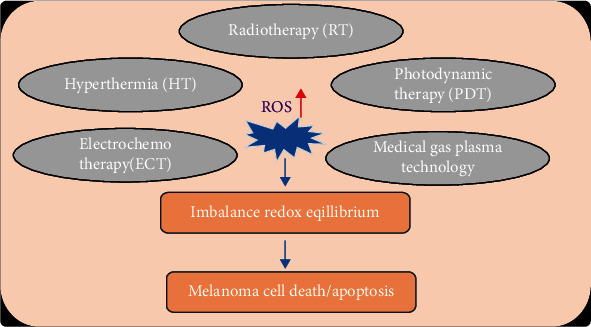
Physical therapy modalities exploiting supraphysiological ROS for melanoma treatment. Several treatments exploit ROS-based effects, including radiotherapy, photodynamic therapy, cryoablation, hyperthermia, and gas plasma technology. ROS induced by these therapies lead to an imbalance in redox equilibrium and subsequently promote melanoma cell death.

The pillars of oncology are surgery, chemotherapy, radiotherapy, and immunotherapy. Especially, the latter three involve the generation of ROS as a byproduct or as a targeted approach to eliminate melanoma. Besides, several physical modalities have emerged throughout the past three decades that come with therapeutic ROS production. A comprehensive review on all present and experimental therapeutic chemotherapeutic, biological, and immunological modalities for melanoma treatment is out of scope of this review. All these approaches are systemic treatments where local control of ROS production may be challenging. Instead, we here focus on local treatments of physical modalities reported to come with augmented ROS production (Figure 6).

### 4.1. Radiotherapy

Radiotherapy is one of the key therapeutic options in oncology. Radiotherapy produces radiation-induced ROS. For instance, OH· is formed directly by the radiolysis of water molecules or indirectly by the formation of secondary ROS [162]. These molecules indiscriminately attack nearby molecules such as DNA and target membranes of cells and organelles, leading to cell cycle arrest and apoptosis [163]. ROS induced by radiation can induce cell death through necrosis, autophagy, mitotic cell death, and cell cycle arrest [163]. The mechanism varies by cell- and tissue-specific factors. ROS generated by radiation triggers DNA damage and apoptosis [162, 163]. Radiotherapy may cause damage to normal tissues alongside tumor. Resistance or sensitivity of tumor cells to radiotherapy depends on cell cycle phase, endogenous antioxidant levels, oxygen availability, and gene expression. Many tumors divide slowly due to their long duration time in the S-phase/interphase of the cell cycle. Therefore, they get more time to repair the damaged DNA, which results in radiation resistance [164]. Several studies showed that mitotic cells with the lowest SH-AOs (SH-containing groups) are most radiosensitive than S-phase cells, which have the highest levels of these compounds [164, 165]. Henceforth, synchronization of the cell cycle under these two sensitive cell cycle phases supports tumor eradication [166]. Moreover, rapidly dividing tumors are more prone to ROS-induced oxidative damage than slowly dividing tumors [165, 167]. In addition, MAPK activation and VEGF release after radiation result in reduced tumor cell response, as shown by several experimental studies [168]. ROS-regulated MAPK determines whether tumor cells proliferate or undergo cell cycle arrest or apoptosis. Lastly, the DNA damage ability of ionizing radiation can be reduced by the antioxidant molecules inside the cells [165]. Several studies have shown that depletion of GSH and its synergistic effects with thioredoxin could increase the radiosensitivity of squamous cell carcinoma cell lines [169]. Intriguingly, depletion of ROS scavengers in cancer stem cells (CSCs) rapidly decreases their clonogenicity and consequence in radiosensitization [170].

The role of NRF2 in radioresistance is well described by recent studies [171, 172]. Aberration of NRF2 activation due to decreased KEAP1-NRF2 interaction and loss of mutations of KEAP1 leads to radiotherapy resistance [172]. In the presence of certain antioxidants, resistant tumor cells respond to radiation-induced killing mostly via ROS-mediated apoptosis [162, 165, 168, 172]. However, there is limited evidence for definitive radiation therapy in melanoma, besides palliation [173]. However, retrospective and phase II studies have divulged that adjuvant radiotherapy can significantly improve the local-regional control rate in a specific clinical setting [174]. Adjuvant radiotherapy is offered to patients who are at high risk of recurrence [175]. Dose and fractionation schedules depend upon the melanoma site, even though the optimal radiation fractionation schedule remains controversial and convenient for patients with low survival expectations. ROS production is a well-recognized mechanism in radiotherapy [176, 177]. This occurs during the tumor treatment and after that, as ROS are being released by stressed and dying cells in the TME due to uncoupled ETC and subsequent superoxide production. Accordingly, antioxidants were found to dampen the efficacy of radiotherapy in preclinical cancer models [178]. Moreover, hypoxic tumors were found to show enhanced radioresistant, and combination treatment with ROS-promoting therapies has been hypothesized to overcome this limitation [179].

### 4.2. Cryoablation

Cryoablation is an intrusive treatment that uses nitrogen or argon gas to create extreme cold to freeze and destroy tumors. The therapy induces tumor cell death by necrosis, hyperosmosis, and apoptosis [180]. Therefore, the treatment is not tightly entangled with the action of ROS [181], but since the therapy is an integral part of melanoma management, it is briefly outlined here nevertheless. The intracellular contents of cryoablation-damaged cells remain preserved for the immune system's recognition to initiate a tumor-specific immune response. Cryoablation slows down the rate of tumor spread and weakens tumor load by ablation of the primary site [182]. Cryoablation combined with distinct immunostimulants enhances the efficacy of cryoablation for the suppression of new tumor growth in metastatic mouse models [183, 184]. This combination can overcome the limitations of immunotherapy. The combination of cryoablation with various immunostimulants (including TLR9 and CPG) has suppressed new tumor growth in metastatic mouse models [184, 185]. In a recent study, cryoablation combined with a transarterial infusion of pembrolizumab has shown promising clinical activity in managing melanoma liver metastasis. However, the efficacy of the therapy needs to be confirmed with a controlled trial in the future [186]. ROS production can be anticipated with this destructive modality, but the therapeutic relevance for cryoablation-related ROS in melanoma remains limited as of now.

### 4.3. Electrochemotherapy

Electrochemotherapy (ECT) is a technique that involves the harmonious use of high-intensity electric pulses to the tumor to increase the cytotoxicity of anticancer drugs, bleomycin and cisplatin, via electroporation [187]. The therapeutic efficacy of electroporation itself without drug application is negligible [188]. The cytotoxicity of drugs such as cisplatin and bleomycin increases by a factor of 100-1000 by electroporation of cell membranes [189]. ECT results demonstrate to be effective for treating cutaneous and subcutaneous malignant melanoma modules [190]. No major negative AEs were observed [191]. ROS production is a byproduct of the pulsed electric field treatment and plays a role in its efficacy [192] as it can be prevented by antioxidants [193, 194]. ROS production and oxidative stress were also observed in electroporated melanoma cells in vitro [195]. Novel ECT approaches involve calcium electroporation with promising clinical results [196]. The treatment engages antitumor immunity to promote systemic attack of metastasis distant to the treatment side [197]. A case report showing such an abscopal effect in a melanoma patient has been published [198]. The calcium treatment locally elevates ROS that contributes to this effect [199, 200] and modulates the tumor vasculature [201]. Hence, this physical treatment modality might be a promising approach for treating therapy-resistant melanoma metastasis, as well as releasing tumor antigen for immunotherapies. A study protocol for a randomized clinical trial in this regard for skin cancer treatment was recently published [202].

### 4.4. Hyperthermia

Hyperthermia is described as the use of exogenous heat sources that directly kill tumor cells or intensify the efficacy of other therapeutic means (e.g., radiotherapy, chemotherapy, and immunotherapies) against various cancer types. Mild hyperthermia as an adjuvant has shown improved antitumor immune response in preclinical and clinical data [203–205]. Hyperthermia generates heat-shock proteins, induces the activation and migration o dendritic cells (DCs), increases the efficacy of tumor antigen presentation, and releases chemo attractants to tumor sites for leukocyte immigration and activation [206]. In vitro studies have shown that hyperthermia inhibits the mobility and proliferative ability of B16F10 cells in a temperature-dependent manner and regulates the TGF-*β*1 protein expression in mouse malignant melanoma B16F10 cells both in vivo and in vitro [207]. In a metastatic mouse model, hyperthermia has significantly extended survival in an animal model. In addition, hyperthermia enhances the therapeutic effectiveness of drugs by activating caspase-8 and caspase-9 to trigger apoptotic responses [208]. The imperative role of ROS in hyperthermia therapy has been thoroughly described [209]. Mechanistically, hyperthermia elevates the levels of transition metal ions, which leads to enhanced production of H_2_O_2_ and OH^·^ by mediating mitochondrial damage. This can be controlled by the amount and duration of heat applied. In doing so, the heat shock also promotes autophagy and local apoptosis [210] while preserving the ability to mount antitumor immunity. Therefore, hyperthermia is well suited to be combined with, e.g., checkpoint therapy. The hyperthermic treatment will generate controlled melanoma cell destruction ROS-dependent and locally restricted without any debulking or therapeutic intent. The tumor antigens subsequently released may then augment antitumor immunity that, in combination with checkpoint therapy, will promote the systemic targeting of melanoma metastasis [211]. This ROS and stress-based heat therapy also show great promise in combination with targeted and nontargeted chemotherapy [208]. Clinical research on hyperthermia in melanoma therapy exists [212], but only a few centers work with techniques so far.

### 4.5. Medical Gas Plasma Technology

Along similar lines, gas plasma technology may be usefully combined with existing oncotherapies [213]. In contrast to hyperthermia, where heat and ROS are generated within the melanoma tissues at sufficient depths, gas plasma technology generates exogenous ROS applied topically to the treatment target [214]. Therefore, it might be well suited in the palliative setting for ulcerating melanoma lesions not covered by skin [215]. Gas plasma technology is unique in generating a plethora of ROS simultaneously with dozens of different agents [216]. The concentration of the ROS can be tuned by changing the ionization variables and the target exposure time [217], while the ambient air condition was found to have a lower impact [218]. As a mechanism of action, gas plasma-derived ROS modulate the expression of redox-regulating enzymes and pathways [219–221] and was found to show combinatorial effects with chemotherapy [222–224], radiotherapy [225–227], and antibody [228] and topical immunotherapy [229]. We have recently also reported for the first time apoptotic effects in patient-derived melanoma tissues [230] and that ROS-derived oxidative posttranslational protein modifications (oxPTMs) generated with gas plasma technology have immunogenic properties and protect from melanoma growth in vivo [231]. Such an approach would be entirely novel in upgrading antitumor vaccines [232] used, for instance, for autologous DC vaccination. Intriguingly, we were also the first to report an abscopal effect in a model of breast cancer where the tumor size of an untreated murine flank decreased in parallel to that of the treated flank, suggesting engagement of antitumor immunity using gas plasma-derived therapeutic ROS [233], as suggested before using human NK cell-mediated melanoma killing in vitro [234]. Gas plasma technology is safe and virtually free of side effects [235–237]. Several devices are marketed in Europe based on accreditation as medical device class IIa [238]. Clinical experience shows promising results in treating actinic keratosis [239–241] and locally advanced head and neck cancer in palliative patients [242–244], and more clinical research is heavily awaited.

### 4.6. Photodynamic Therapy

Photodynamic therapy (PDT) has a long-standing application in the clinic already. This light-based and minimally invasive therapy is promising and effective in various types of cancers including nonmelanoma and melanoma skin cancer, for patients with stage III/IV cutaneous metastatic melanomas [245]. PDT is a minimally invasive procedure [246] that requires a photosensitizer (PS) molecule which, upon excitation by the specific wavelength of light, reacts with oxygen and causes oxidant species in target tissues, leading to increased ROS production, redox signaling, and cell death [247]. The advantage of PDT is its low systemic toxicity and its ability to destroy tumors selectively [246]. ROS, especially singlet oxygen, unleash irreversible damage to tumor cells and tumor-associated blood vessels, also activating antitumor immunity via inflammatory responses [248]. The limited penetration of light restricts the clinical use of PDT. For better efficacy of PDT in melanoma, overcoming protective mechanisms such as pigmentation and oxidative stress resistance is necessary to treat intracutaneous lesions [249]. The concepts and challenges of oxidative stress and PDT in skin cancer have been elegantly reported recently [250]. Clinically, PDT can be used along with other procedures, such as surgery, radiotherapy, or chemotherapy [251, 252]. Combined therapies have been studied to overcome melanoma resistance. The combination of PDT and chemotherapy (dacarbazine) was an efficient treatment to overcome the internal resistance in metastatic melanoma [253]. Most ongoing trials for cancer are using the photosensitizers that are approved for clinical use, mainly ALA (aminolevulinic acid) and photofrin (porfimer sodium) [254]. Combining PDT with immunoadjuvants to stimulate the antitumor immunity was more efficient and safer for treating melanoma than the monotherapies strategy [255], but still needs more clinical studies to elaborate efficacy and safety.

## 5. Conclusion

**Figure 7:**
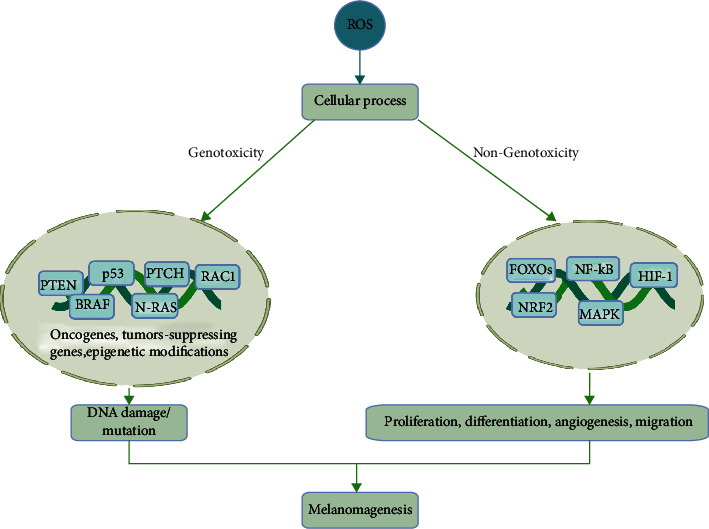
ROS role in melanomagenesis. High ROS levels activate protooncogenes, inactivate tumor suppressor genes, and cause genetic modifications. This leads to DNA damage and mutations and involves cellular signaling that blunts DNA repair and cell death and drives melanomagenesis. Conversely, signaling pathways less involved in DNA repair, such as MAPK, NF-*κ*B, PI3K/AKT/mTOR, and NRF2 spur melanoma cells' proliferation, angiogenesis, and metastasis. Together, these processes cause the occurrence of melanoma. Created with http://biorender.com.

**Figure 8:**
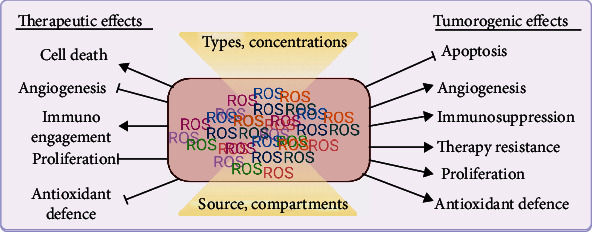
Pleitropic roles of ROS in melanoma. ROS have pleitropic roles in melanoma therapy and tumorigenesis depending on their source, concentration, location, and types. Created with http://biorender.com.

Following a review of ROS' divergent biological processes, some generalizations regarding the induction and function of ROS can be made. Importantly, ROS are generally induced by cell stress, starvation, hypoxia, and growth factor stimulation. Mild ROS induction promotes adaptation to ROS stress via HIF activation under hypoxia, inflammatory cytokine production in tissue damage, and differentiation in receptor-dependent stimulation, conjointly promoting cell survival (Figure 7). Although ROS have been recognized as important second messengers in cell biology, they have only recently gained attention concerning melanoma biology. At higher levels, ROS are detrimental, and several physical modalities directly or indirectly exploit such damaging functions for the treatment of skin cancer (Figure 8), including melanoma. As primary and metastatic melanoma provides high dynamic and plasticity in the TME, specifically targeting ROS is challenging as melanoma cells adapt to altered redox environments.

Even though the failure of dietary antioxidants in several clinical trials resulted in the emergence of alternative antimelanoma therapeutic approaches, there is still considerable controversy as to whether the use of either antioxidant supplementation or inhibition of ROS modulation is detrimental or beneficial for melanoma treatment. The effect of ROS is predominantly near the site of ROS production. Therefore, the use of inhibitors or antioxidants may prevent melanomagenesis. In addition to that, prooxidant melanoma therapy, through which scavenging of ROS is decreased or production of ROS is increased, or both, is a pioneering approach to exploit the higher levels of ROS in cancer cells to elicit cell death selectively. The eminent role of ROS modulation in antimelanoma therapies like ROS-inducing drugs and physical modalities, such as radiotherapy and photodynamic therapy, is well reported. It follows that combination therapies, e.g., checkpoint inhibition and ROS-generating therapies, might constitute novel avenues for targeting melanoma. While more preclinical and clinical research is excitingly awaited, the data known to date are encouraging that understanding, targeting, and utilizing ROS in melanoma treatment might be an effective adjuvant treatment in patient therapy in the future.

## Abbreviations

CAFs: Cancer-associated fibroblasts
CAT: Catalase
ECM: Extracellular matrix
ECT: Electrochemotherapy
ETC: Electron transport chain
GPX: Glutathione peroxidase
GSH: Glutathione
GSS: Glutathione synthetase
GSSG: Oxidized glutathione
H_2_O_2_: Hydrogen peroxide
HIF: Hypoxia-inducible factor
IL: Interleukin
KEAP1: Kelch-like ECH-associated protein 1
MMP: Matrix metalloproteinases
NADPH: Nicotinamide adenine dinucleotide phosphate
NF-*κ*B: Nuclear factor kappa-light-chain-enhancer of activated B cells
NOS: Nitric oxide synthase
NOX: NADPH oxidase
NRF2: Nuclear factor erythroid 2-related factor 2
O_2_^−^: Superoxide ion
OH·: Hydroxyl radical
PDT: Photodynamic therapy
PRDX: peroxiredoxins
ROS: Reactive oxygen species
SOD: Superoxide dismutase.
